# Surgery and chemotherapy cannot improve the survival of patients with early-stage mucosa-associated lymphoid tissue derived primary pulmonary lymphoma

**DOI:** 10.3389/fonc.2022.965727

**Published:** 2022-08-23

**Authors:** Huahang Lin, Ke Zhou, Zhiyu Peng, Linchuan Liang, Jie Cao, Jiandong Mei

**Affiliations:** ^1^ Department of Thoracic Surgery, West China Hospital, Sichuan University, Chengdu, China; ^2^ Western China Collaborative Innovation Center for Early Diagnosis and Multidisciplinary Therapy of Lung Cancer, Sichuan University, Chengdu, China

**Keywords:** early-stage, mucosa-associated lymphoid tissue, primary pulmonary lymphoma, propensity score-matching analysis, survival, treatment modalities

## Abstract

**Background:**

To date, there is no treatment consensus on mucosa-associated lymphoid tissue (MALT) derived primary pulmonary lymphoma (PPL).

**Methods:**

We identified patients with early-stage MALT-type PPL from the National Cancer Institute’s Surveillance, Epidemiology, and End Results program database. The patients were divided into four groups according to treatment modalities: None of surgery or chemotherapy (None) group, Surgery alone group, Chemotherapy alone (Chemo alone) group, and Surgery plus chemotherapy (Surgery + chemo) group. Overall survival (OS) and cancer-specific survival (CSS) were study endpoints. We performed Cox regression analyses, propensity score-matched analyses (PSM) and Kaplan-Meier (KM) survival curves to compare the survival among different groups.

**Results:**

A total of 953 patients were included in our analysis with 302, 403, 175, and 73 cases in the None, Surgery alone, Chemo alone, and Surgery + chemo groups, respectively. In this cohort, the estimated 3-year, 5-year and 10-year OS rates were 86.95%, 78.91%, and 55.89%, respectively. Meanwhile, the estimated 3-year, 5-year and 10-year CSS rates were 96.71%, 93.73%, and 86.84%, respectively. Multivariate Cox regression analyses demonstrated that increasing age, tumors located in the lower lobe, and stage II were significant predictors of poorer OS while increasing age and tumors located in the bilateral lungs were associated with lower CSS. After PSM analyses, the KM survival curves showed no significant differences in OS or CSS among the four groups.

**Conclusion:**

Early-stage MALT-type PPL is indolent in nature. Neither surgery, chemotherapy nor a combination of surgery and chemotherapy can improve OS and CSS, suggesting that “watch and wait” may be a reasonable alternative.

## Introduction

Primary pulmonary lymphoma (PPL) refers to lymphomas occurring primarily from the lungs representing 0.4% of all lymphomas, 3–4% of extranodal lymphoma, and only 0.5%-1% of primary pulmonary neoplasms (1, 2). PPL is defined as a malignant clonal proliferation of lymphatic tissue in the lung, which must be differentiated from the metastasis to lung of lymphoma from other sites, such as stomach. In 1993, Cordier developed the following diagnostic criteria for PPL (3) (1) definite pathological evidence showing pulmonary lymphoma (2) chest X-ray showing the lesions in the unilateral or bilateral lungs without evidence of mediastinal adenopathy or mass, or computed tomography (CT) scan showing predominant pulmonary involvement with mediastinal adenopathy (3) no extrathoracic lymphoma previously diagnosed (4) no extrathoracic lymphoma or lymphatic leukemia detected at diagnosis (5) no detectable extrathoracic involvement at diagnosis or in the next 3 months.

The most common type of PPL is the mucosa-associated lymphoid tissue (MALT) derived lymphoma followed by diffuse large B-cell lymphoma (DLBCL), and these two types of lymphomas account for 90% of PPL (4, 5). A survey showed an 18% increase in the incidence of MALT-type PPL between 2006 and 2009 compared with 2001 and 2005, indicating that the number of the patients is gradually increasing (6). MALT-type lymphoma was first described by Isaacson and Wright in 1983, and can be seen in a variety of organs containing epithelial tissues, such as the stomach, salivary gland, lung, and small intestine (7). The gastrointestinal tract is the most common site of involvement, with lung involvement accounting for 15% (8). Due to the low incidence of MALT-type PPL, lack of specificity in clinical and radiological manifestations, and insufficient understanding of the disease, it was difficult to give a definite diagnosis, leading to a high misdiagnosis rate (9). Fortunately, it was widely admitted that MALT-type PPL has a favorable prognosis because of its indolent nature (10–12). To date, there is no consensus concerning the treatment of MALT-type PPL. The regimens included “watch and wait”, surgery, chemotherapy, radiotherapy, immunotherapy and so on, which could be used alone or in combination (13–16).

Given the lack of analysis of population-based data and consensus on treatment, this study attempted to compare the efficacy of different treatment modalities on early-stage MALT-type PPL using the National Cancer Institute’s Surveillance, Epidemiology, and End Results (SEER) program database.

## Methods

### Database and patients’ selection

Clinical data on patients with MALT-type PPL were extracted from the SEER database with 18 registries between 1983 and 2015. The criteria for eligibility in this analysis were as follows (1) patients were diagnosed with Ann Arbor stage I/II primary pulmonary MALT lymphoma between 1983 and 2015; and (2) MALT-type PPL was pathologically proven. Patients who met the following criteria were excluded: (I) patient survival was unknown; (II) patients were considered as secondary lymphoma; and (III) patients receiving radiotherapy were excluded from this analysis. Notably, we excluded those patients treated with radiotherapy because radiotherapy was rarely prescribed to treat early-stage MALT-type PPL. A total of 1013 patients in Ann Arbor stage I or II were retrieved using the International Classification of Diseases for Oncology Version 3 (ICD-O-3) histology codes 9699, and primary site codes C34.0, C34.1, C34.2, C34.3, C34.8 and C34.9. Cases without available survival information (n = 10), proven to be secondary lymphoma (n = 23), and receiving radiotherapy (n = 27) were excluded. Finally, we enrolled 953 cases in our study.

### Treatment strategies

The treatments for MALT-type PPL comprised of “watch and wait”, surgery, chemotherapy, radiotherapy and immunotherapy. “Watch and wait” meant that patients did not receive any treatment except for regular follow-up. In our study, 152 patients underwent wedge resection or segmentectomy, 300 patients underwent lobectomy or pneumonectomy, and no information on the type of surgical procedure was provided in 24 patients. Concerning chemotherapy, the specific regimens were unknown in the SEER database. Radiotherapy was reserved only for patients with a unique, small lesion in a poorly mobile site and with contraindications to surgery (17). Information on immunotherapy could not be obtained in the SEER database. Therefore, we mainly focused on the analysis of the efficacy of “watch and wait”, surgery, and chemotherapy.

### Variables and outcomes

The following data were extracted from each case: age at diagnosis, sex, race, marital status, year of diagnosis, primary site, laterality, Ann Arbor stage, treatments, survival time, survival status, and cause of death. The stage of lymphoma was determined according to Ann Arbor staging instead of American Joint Committee on Cancer (AJCC) staging (4, 18).

The outcomes in this study included overall survival (OS) and cancer-specific survival. The SEER cause of death was categorized as cancer-specific death, other-cancer death, chronic pulmonary disease, heart disease, and others. OS was defined as the time from diagnosis to either death or last follow-up. CSS was defined as the time from diagnosis to death from primary pulmonary lymphoma, or last follow-up.

### Statistical analysis

The patients were divided into four groups according to treatment modalities: None of surgery or chemotherapy (None) group, Surgery alone group, Chemotherapy alone (Chemo alone) group, and Surgery plus chemotherapy (Surgery + chemo) group. In the comparison of baseline characteristics among the four groups, continuous variables were tested by t test and categorical variables were tested by Chi-square or Fisher’s exact test. Unadjusted OS and CSS estimates of the entire cohort were obtained by Kaplan-Meier (KM) survival curve plotting. Cox regression analyses were used to identify the variables associated with survival. Univariate Cox regression analyses were initially performed to recognize the significant variables and then they were further analyzed in multivariate Cox regression analyses. Propensity score-matched (PSM) analyses were performed by using the 1:1 nearest neighbor technique with a small caliper of 0.05 to balance the characteristics among the four groups, including age, sex, race, marital status, year of diagnosis, primary site, laterality and Ann Arbor stage. After PSM, adjusted KM survival curves for OS and CSS were drawn again to compare the survival among the four groups in pairs. All these analyses were conducted by SPSS software version 22.0 and R software version 4.1. The significance level was set to two-tailed P < 0.05 for all analyses.

## Results

### Patient characteristics

**Figure 1:**
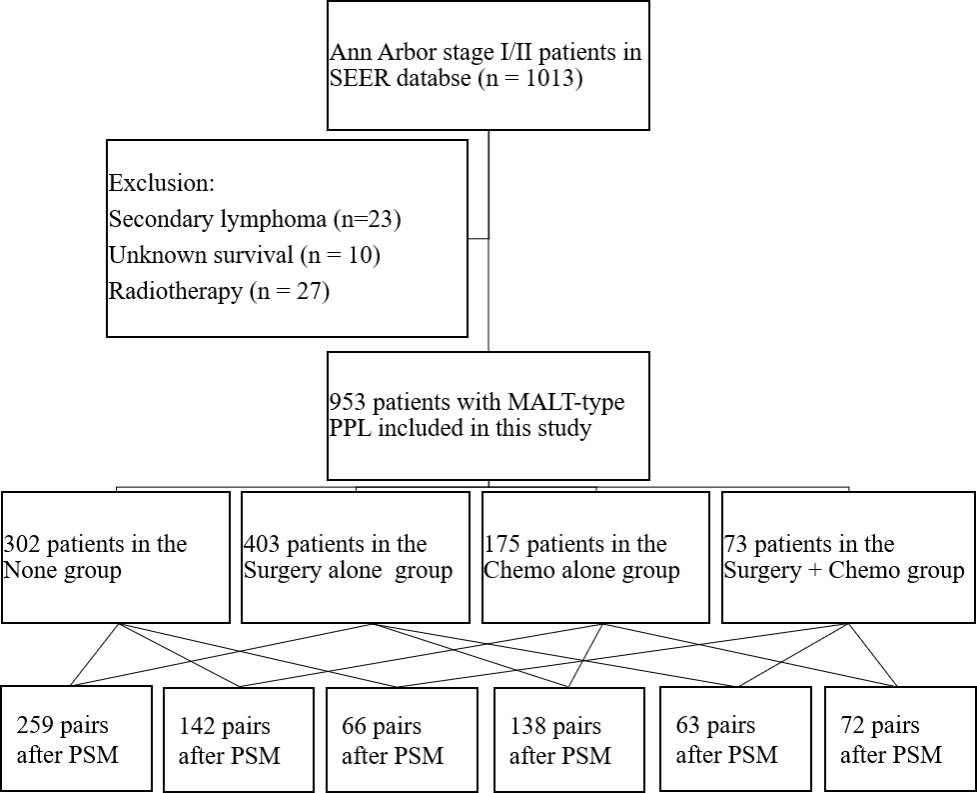
The schema and distribution of the study population. PSM: propensity score matching.

A total of 953 patients suffering from early-stage MALT-type PPL were retrospectively reviewed in this study (**Figure 1**). The clinicopathological characteristics of the patients are shown in **Table 1**. In the entire cohort, the average age was 67.23 ± 12.05 years old with a female: male ratio of approximately1.44:1 (59.1% vs 40.9%). For most of the patients, tumors located in specific sites (79.3%) and in unilateral lung (90.9%). In addition, the majority of cases belonged to stage I (82.8%). In terms of the treatment modalities, the distribution of the study population was as follows: 302 patients in the None group (31.7%), 403 patients in the Surgery alone group (42.3%), 175 patients in the Chemo alone group (18.4%), and 73 patients in the Surgery + Chemo group (7.6%). Among the four groups, the variables were well balanced with regard to race, sex and marital status. However, age, year at diagnosis, primary site, laterality and stage were not comparable.

**Table 1:** Characteristics of primary pulmonary MALT lymphoma among the four groups.

Variables		Total	None	Surgery alone	Chemo alone	Surgery + Chemo	P value
Distribution		953	302	403	175	73	
Age(years)		67.23 ± 12.05	69.78 ± 11.89	65.92 ± 11.54	67.66 ± 12.76	62.89 ± 11.67	<0.001
Sex	Female	563 (59.1%)	165 (54.6%)	252 (62.5%)	103 (58.9%)	43 (58.9%)	0.216
	Male	390 (40.9%)	137 (45.4%)	151 (37.5%)	72 (41.1%)	30 (41.1%)	
Race	White	818 (85.8%)	255 (84.4%)	351 (87.1%)	144 (82.3%)	68 (93.2%)	0.188
	black	72 (7.3%)	22 (7.3%)	31 (7.7%)	15 (8.6%)	4 (5.5%)	
	Other	63 (6.6%)	25 (8.3%)	21 (5.2%)	16 (9.1%)	1 (1.4%)	
Marital status	Single	49 (15.4%)	49 (16.2%)	60 (14.9%)	29 (16.6%)	9 (12.3%)	0.401
	Married	174 (60.1%)	174 (57.6%)	241 (59.8%)	105 (60.0%)	53 (72.6%)	
	Separated	233 (24.4%)	79 (26.2%)	102 (25.3%)	41 (23.4%)	11 (15.1%)	
Year of diagnosis	<2000	19 (6.8%)	19 (6.3%)	36 (8.9%)	6 (3.4%)	4 (5.5%)	<0.001
	2000-2009	458 (48.1%)	112 (37.1%)	210 (52.1%)	89 (50.9%)	47 (64.4%)	
	≥2010	430 (45.1%)	171 (56.6%)	157 (39.0%)	80 (45.7%)	22 (30.1%)	
Primary site	Upper lobe	305 (32.0%)	93 (30.8%)	141 (35.0%)	56 (32.0%)	15 (20.5%)	<0.001
	Middle lobe	123 (12.9%)	32 (10.6%)	66 (16.4%)	17 (9.7%)	8 (11.0%)	
	Lower lobe	314 (32.9%)	98 (32.5%)	156 (38.7%)	41 (23.4%)	19 (26.0%)	
	Main bronchus	14 (1.5%)	4 (1.3%)	2 (0.5%)	6 (3.4%)	2 (2.7%)	
	NOS	197 (20.7%)	75 (24.8%)	38 (9.4%)	55 (31.4%)	29 (39.7%)	
Laterality	Unilateral	866 (90.9%)	269 (89.1%)	393 (97.5%)	149 (85.1%)	55 (75.3%)	<0.001
	Bilateral	87 (9.1%)	33 (10.9%)	10 (2.5%)	26 (14.9%)	18 (24.7%)	
Stage	I	789 (82.8%)	258(85.4%)	356 (88.3%)	115 (65.7%)	60 (82.2%)	<0.001
	II	164 (17.2%)	44 (14.6%)	47 (11.7%)	60 (34.3%)	13 (17.8%)	

NOS, not other specified.

### Survival

At a median follow up of 64 months, the median OS was 144 months (95% CI 127.30-160.70 months), and the median CSS was not reached. Unadjusted OS and CSS estimates at 3, 5, and 10 years are summarized in **Table 2** according to treatment modalities. In this cohort, the estimated 3-year, 5-year and 10-year OS rates were 86.95%, 78.91%, and 55.89%, respectively. Meanwhile, the estimated 3-year, 5-year and 10-year CSS rates were 96.71%, 93.73%, and 86.84%, respectively. At a rough estimate from **Table 2**, patients undergoing surgery were associated with superior survival, and patients receiving chemotherapy showed inferior survival.

**Table 2:** Unadjusted Survival Estimates.

Group	Overall survival			Cancer-specific survival		
	3-year	5-year	10-year	3-year	5-year	10-year
Total	86.95% (95% CI 84.56%-89.00%)	78.91% (95% CI 75.92%-81.57%)	55.89% (95% CI 51.50%-60.04%)	96.71% (95% CI 95.26%-97.72%)	93.73% (95% CI 91.70%-95.27%)	86.84% (95% CI 83.25%-89.70%)
None	83.06% (95% CI 78.11%-86.98%)	74.50% (95% CI 68.45%-79.55%)	48.24% (95% CI 39.07%-56.80%)	95.50% (95% CI 92.17%-97.43%)	93.63% (95% CI 89.46%-96.18%)	85.85% (95% CI 77.31%-91.36%)
Surgery alone	90.96% (95% CI 87.57%-93.46%)	84.86% (95% CI 80.61%-88.25%)	60.60% (95% CI 54.17%-66.42%)	98.28% (95% CI 96.19%-99.22%)	96.16% (95% CI 93.30%-97.81%)	89.20% (95% CI 84.04%-92.76%)
Chemo alone	81.47% (95% CI 74.68%-86.60%)	71.65% (95% CI 63.83%-78.07%)	47.91% (95% CI 37.19%-57.84%)	95.01% (95% CI 90.23%-97.48%)	90.79% (95% CI 84.51%-94.60%)	79.99% (95% CI 67.12%-88.25%)
Surgery + Chemo	94.13% (95% CI 85.11%-97.76%)	81.69% (95% CI 69.97%-89.17%)	70.75% (95% CI 56.58%-81.04%)	97.03% (95% CI 88.63%-99.25%)	88.94% (95% CI 78.14%-94.58%)	88.94% (95% CI 78.14%-94.58%)

CI, confidence interval.

**Figure 2:**
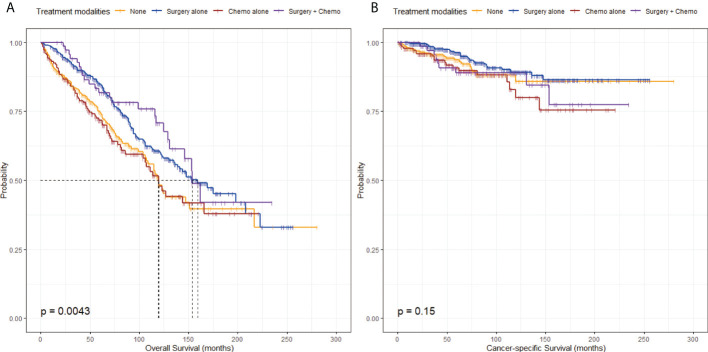
Kaplan-Meier survival curves stratified by treatment modalities before propensity score-matching. **(A)** Kaplan-Meier survival curve of overall survival stratified by treatment modalities. **(B)** Kaplan-Meier survival curve of cancer-specific survival stratified by treatment modalities.

The unadjusted KM survival curves for OS and CSS among the four groups in **Figure 2** revealed that there were significant differences in OS (P = 0.0043) but not in CSS (P = 0.15). From the KM survival curve for OS, the relatively excellent OS was shown in the Surgery alone group or the Surgery + Chemo group graphically (**Figure 2**).

**Table 3** shows the variables associated with survival. In the Cox regression analysis for OS, several characteristics were incorporated into the multivariate Cox regression analysis after univariate Cox regression analysis, including age, marital status, primary site, stage, and treatment modalities. The results of the multivariate Cox regression analysis demonstrated that increasing age (HR = 1.077, 95% CI: 1.061-1.093), tumors located in the lower lobe (HR = 0.652, 95% CI: 0.454-0.936), and stage II disease (HR = 1.494, 95% CI: 1.008-2.213) were independent clinical predictors of poorer OS. With regard to CSS, we included age, marital status, year of diagnosis, and laterality in the multivariate Cox regression analysis. Then it was obvious that increasing age (HR = 1.031, 95% CI: 1.008-1.055) and tumors located in the bilateral lungs (HR = 2.655, 95% CI: 1.336-5.275) could lead to lower CSS. From the Cox regression analyses, we identified that the treatment modalities were not correlated with the survival.

**Table 3:** Univariate and multivariate Cox regression analyses of overall survival and disease-specific survival.

Parameters	Overall survival				Cancer-specific survival			
	Univariate analysis		Multivariate analysis		Univariate analysis		Multivariate analysis	
	HR (95% CI)	P value	HR (95% CI)	P value	HR (95% CI)	P value	HR (95% CI)	P value
Age(years)	1.080 (1.067-1.093)	<0.001	1.077 (1.061-1.093)	<0.001	1.049 (1.027-1.073)	<0.001	1.031 (1.008-1.055)	0.008
Sex								
Female	Reference				Reference			
Male	1.001 (0.801-1.252)	0.990			1.226 (0.782-1.919)	0.374		
Race								
White	Reference				Reference			
black	0.674 (0.428-1.061)	0.088			0.858 (0.372-1.980)	0.720		
Other	0.742 (0.448-1.228)	0.246			0.388 (0.095-1.582)	0.187		
Marital status								
Single	Reference		Reference		Reference		Reference	
Married	0.954 (0.683-1.332)	0.782	0.898 (0.585-1.380)	0.624	1.399 (0.628-3.118)	0.412	1.235 (0.532-2.867)	0.623
Separated	1.681 (1.178-2.398)	0.004	1.205 (0.744-1.952)	0.449	2.809 (1.230-6.413)	0.014	1.942 (0.795-4.740)	0.145
Year of diagnosis								
<2000	Reference				Reference		Reference	
2000-2009	1.030 (0.727-1.460)	0.868			0.745 (0.396-1.399)	0.359	0.573 (0.282-1.165)	0.573
≥2010	0.753 (0.489-1.158)	0.196			0.351 (0.150-0.820)	0.016	0.107 (0.044-0.260)	0.107
Primary site								
Upper lobe	Reference		Reference		Reference			
Middle lobe	0.704 (0.492-1.008)	0.055	0.782 (0.484-1.262)	0.314	0.872 (0.401-1.894)	0.728		
Lower lobe	0.625 (0.474-0.824)	0.001	0.652 (0.454-0.936)	0.020	0.871 (0.482-1.573)	0.646		
Main bronchus	1.322 (0.645-2.710)	0.445	3.551 (0.981-12.855)	0.054	2.914 (0.870-9.759)	0.083		
NOS	0.841 (0.623-1.136)	0.259	0.944 (0.621-1.433)	0.786	1.459 (0.802-2.653)	0.216		
Laterality								
Unilateral	Reference				Reference		Reference	
Bilateral	1.061 (0.871-1.292)	0.557			1.980 (1.090-3.594)	0.025	2.655 (1.336-5.275)	0.005
Stage								
I	Reference		Reference		Reference			
II	1.345 (1.026-1.764)	0.032	1.494 (1.008-2.213)	0.046	1.586 (0.936-2.689)	0.087		
Treatment modalities								
None	Reference		Reference		Reference			
Surgery alone	0.703 (0.541-0.914)	0.008	1.298 (0.911-1.850)	0.149	0.695 (0.395-1.222)	0.206		
Chemo alone	1.058 (0.778-1.440)	0.718	1.341 (0.870-2.066)	0.183	1.350 (0.731-2.496)	0.338		
Surgery +Chemo	0.615 (0.393-0.961)	0.033	1.639 (0.901-2.981)	0.106	1.093 (0.502-2.382)	0.823		

CI, confidence interval; NOS, not other specified.

### Propensity score-matched analysis

**Figure 3:**
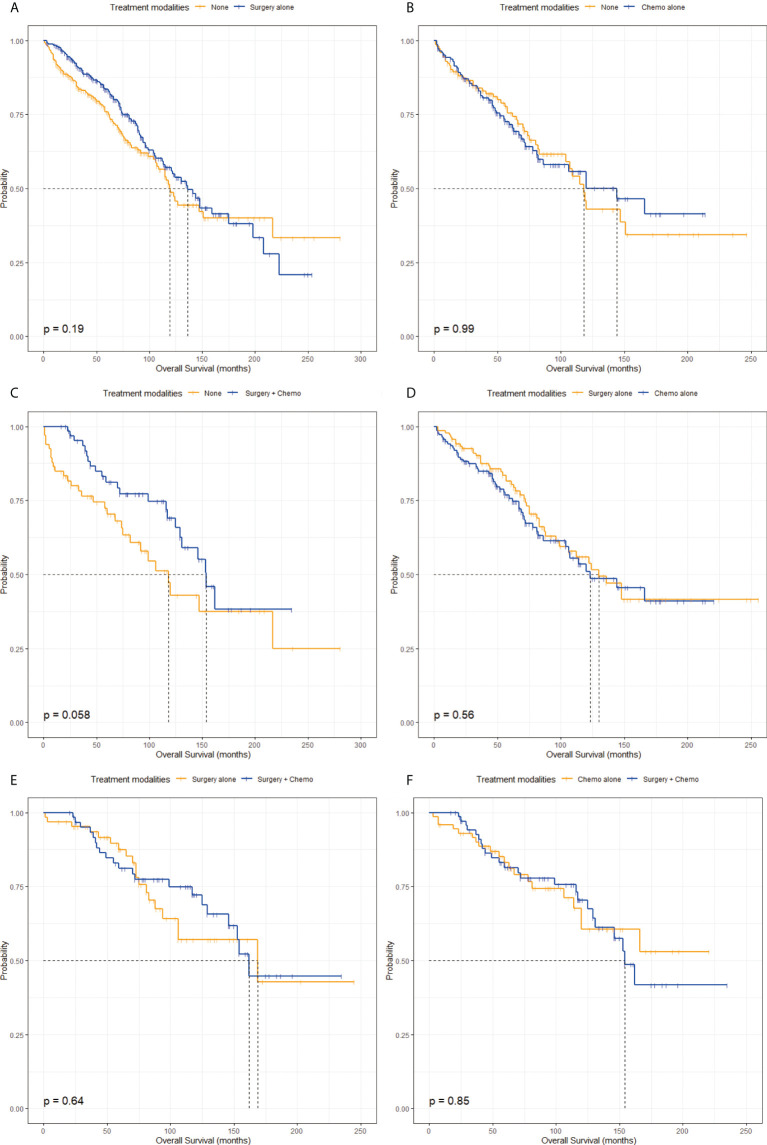
Kaplan-Meier survival curves of overall survival stratified by treatment modalities after propensity score-matching. **(A)** Kaplan-Meier survival curve of overall survival between the None group and the Surgery alone group. **(B)** Kaplan-Meier survival curve of overall survival between the None group and the Chemo alone group. **(C)** Kaplan-Meier survival curve of overall survival between the None group and the Surgery + Chemo group. **(D)** Kaplan-Meier survival curve of overall survival between the Surgery alone group and the Chemo alone group. **(E)** Kaplan-Meier survival curve of overall survival between the Surgery alone group and the Surgery + Chemo group. **(F)** Kaplan-Meier survival curve of overall survival between the Chemo alone group and the Surgery + Chemo group.

**Figure 4:**
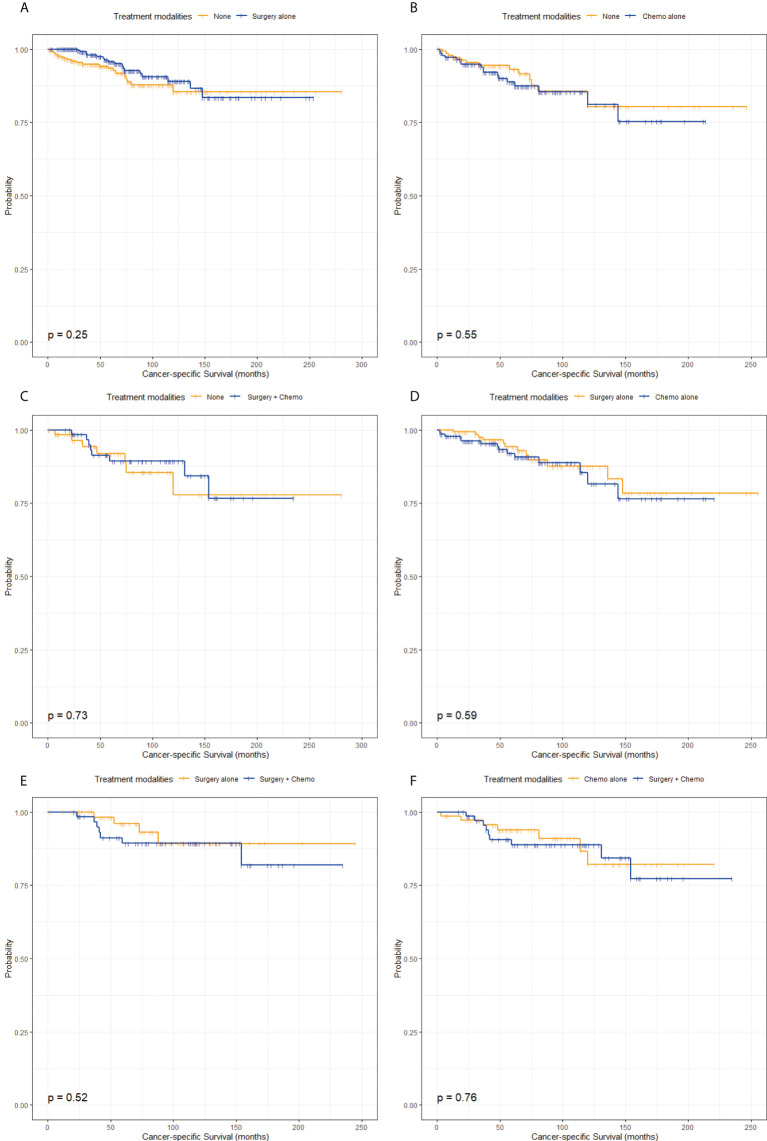
Kaplan-Meier survival curves of cancer-specific survival stratified by treatment modalities after propensity score-matching. **(A)** Kaplan-Meier survival curve of cancer-specific survival between the None group and the Surgery alone group. **(B)** Kaplan-Meier survival curve of cancer-specific survival between the None group and the Chemo alone group. **(C)** Kaplan-Meier survival curve of cancer-specific survival between the None group and the Surgery + Chemo group. **(D)** Kaplan-Meier survival curve of cancer-specific survival between the Surgery alone group and the Chemo alone group. **(E)** Kaplan-Meier survival curve of cancer-specific survival between the Surgery alone group and the Surgery + Chemo group. **(F)** Kaplan-Meier survival curve of cancer-specific survival between the Chemo alone group and the Surgery + Chemo group.

The characteristics in the matched groups were well-balanced after propensity score-matched analyses as shown in **Figure 1** and **Tables S1-6**. From the pairwise comparisons of KM survival curves among the four groups in **Figure 3** and **Figure 4**, it was apparent that regardless of OS or CSS, no significant differences existed (P > 0.05) in the six matching populations. These statistics further indicated that patients in the None, Surgery alone, Chemo alone, and Surgery + Chemo groups had similar long-term OS and CSS.

## Discussion

To our knowledge, this analysis has been the largest population-based series to investigate the treatment strategies for early-stage MALT-type PPL. From our work, it was easily observed that MALT-type PPL considerably increased in the past two decades, which indicated that it was imperative to conduct deeper research on MALT-type PPL. Similar to favorable outcomes previously mentioned in the literatures (5, 14, 15, 19), our results reinforced the favorable outcome of early-stage MALT-type PPL with high OS and CSS rates. After Cox regression analyses, we found that increasing age, tumor located in the lower lobe, and stage II were independent predictors of poorer OS while increasing age and tumors located in the bilateral lungs were associated with lower CSS. To minimize the chance for error and bias, PSM analyses were used to balance the variables among the groups and we found that patients treated with surgery, chemotherapy or a combination of surgery and chemotherapy did not show improved OS and CSS.

In 1963, Saltzstein and his colleague first described the concept of “pseudolymphoma” in the lung (20). However, since the perception of MALT and advances in diagnostic techniques such as immunohistochemistry and gene detection, most of those “pseudolymphomas” were considered to be MALT-type lymphoma (21). Bienenstock first described the existence of pulmonary MALT in 1973 (22). It is generally accepted that human bronchial mucosa is similar to gastric mucosa under normal circumstances, and MALT usually does not exist. However, acquired MALT mucosa could be induced by various antigenic stimulations such as smoking, infection and autoimmune diseases, which may lead to MALT-type PPL (23, 24).

In our analysis, we adopted Ann Arbor staging system because it has been widely used in the clinical staging of PPL (25). It was reported that MALT-type PPL tended to be in early stages (I-II), which was why we focused on early-stage MALT-type PPL (25, 26). The treatments for MALT-type PPL comprised of “watch and wait”, surgery, chemotherapy, radiotherapy and immunotherapy (13–16). However, the optimal treatment is still controversial.

In recent years, an increasing number of studies have stated that “watch and wait” could be an option for early-stage MALT-type PPL. One retrospective analysis identified 11 patients with MALT-type PPL who did not receive any treatment from initial diagnosis. Ultimately, all patients were alive with 3 patients experiencing progression and 8 patients still being watched. It is worth mentioning that the tumors spontaneously regressed or waxed and waned in six of the eleven patients (13). Likewise, a recent study enrolled 14 patients in the “watch and wait” cohort, and 14 were immediately treated with rituximab single agent or combined chemotherapy (R/R-Chemo). The estimated median time of OS was not statistically significant between the “watch and wait” cohort and the immediate R/R-Chemo cohort, with values of 78 months and 76 months, respectively (27). Although we could not recognize whether “watch and wait” or immunotherapy was given to those not treated with chemotherapy or surgery in our work, we deduced that a majority of these patients underwent “watch and wait” remedy on account of infrequent utilization of immunotherapy for MALT-type PPL (17). Thus, our results that patients in the None group did not show inferior OS and CSS indicated that “watch and wait” was a rational choice to a great extent. Furthermore, we built this perspective on the basis of the indolent nature and phenomenon of spontaneous regression of MALT-type PPL (28, 29).

In the management of MALT-type PPL, surgery was considered to be an effective option. A study on B-cell PPL suggested that surgery should be preferred if complete resection can be achieved. Through complete resection, patients with confined PPL could reach excellent long-term survival rates of approximately 90% (30). In a cohort of 43 patients diagnosed with stage I/II MALT-type PPL, 33 patients underwent complete surgical resection and 10 patients did not undergo surgical resection. Consequently, the proportion of patients reaching complete remission was higher in patients undergoing complete surgical resection (97.0% [32/33] vs. 50.0% [5/10], P < 0.001). In addition, postoperative complications (chylothorax and prolonged air leakage) occurred in only two patients. As a result, the authors believed that surgical resection was the mainstay treatment for early-stage MALT-type lymphoma (31). In contrast, a previous study of 38 early-stage MALT lymphoma patients showed no significant difference in OS between patients who underwent surgery and those who did. However, patients treated with chemotherapy had better progression-free survival than those treated with surgery (32). Although the most common treatment was surgery in our analysis, it showed no benefit to OS and CSS. In our opinion, the role of surgery on MALT lymphoma may lie in obtaining adequate tissue for correct diagnosis if it is difficult to clarify the diagnosis (1).

A variety of chemotherapeutic agents, either single or combined, have been widely used in the treatment of MALT-type PPL, but no standard regimen has been established. Zinzani et al. reviewed 31 patients suffering from Stage IE MALT-type lymphoma who accepted either fludarabine/mitoxantrone (FM) or cyclophosphamide, vincristine and prednisone (CVP). In their series, the estimated OS and disease-free survival rates reached 100% and 85%, respectively, at 60 months. In addition, they also found that the fludarabine-containing FM regimen was superior to CVP in terms of efficacy for patients with non-gastrointestinal stage IE MALT lymphoma (33). In 2009, Borie et al. reported that patients with MALT lymphoma receiving chemotherapy with chlorambucil, cyclophosphamide and anthracycline/fludarabine were associated with 3-year progression-free survival rates of 75%, 40%, and 25%, respectively (10). Overall, it was generally admitted that chemotherapy including chlorambucil, CHOP (cyclophosphamide, doxorubicin, vincristine, prednisone), CHOP-like or fludarabine-containing regimens should be the first-line remedy for MALT-type PPL (17). Nevertheless, our results demonstrated that chemotherapy failed to bring better OS or CSS for early-stage MALT-type PPL. In addition, adjuvant chemotherapy followed by radical resection may not provide additional survival benefits (1). Our results also demonstrated that surgery plus chemotherapy could not improve survival.

Radiotherapy with moderate-dose has been reported to provide excellent clinical outcomes for extranodal MALT-type lymphomas (34, 35). However, for cases with MALT-type PPL the outcomes were not as good as MALT-type lymphomas of other sites. Part of the answer may be the fact that the lungs are mobile organs (36). Radiotherapy should be reserved for MALT-type patients with localized lesions in poorly mobile sites and with contraindications to surgery (17). Owing to the limited number of patients treated with radiotherapy, we did not perform an analysis to clarify its role.

Immunotherapy has been widely applied in the therapy of MALT-type lymphomas in different extranodal organs (37, 38). However, there are few studies on its application to MALT-type PPL. A case series reported eight MALT-type PPL patients treated with systemic rituximab monotherapy (375 mg m−2 day−1, 4–8 cycles), of which 6 patients were in stage I/II and 2 were in stage IV. Among them, five patients achieved a complete response, one achieved a partial response, and two were in a stable condition (39). This study showed a bright future of immunotherapy in the therapy of MALT-type PPL. Regretfully, we had no access to immunotherapy information.

We admitted several limitations to our work. First, because of the lack of some clinical characteristics in the SEER database, including tumor size, number of tumors and laboratory measurements, a certain degree of bias may be introduced into the Cox regression analyses and propensity score-matching analysis. Furthermore, the specific chemotherapy plans were unclear, and thus we were unable to separately evaluate the efficacy of different chemotherapy regimens. In addition, it was unknown if those patients not treated with surgery or chemotherapy received “watch and wait” or immunotherapy. Despite these shortcomings in our analysis, we believe that this analysis will provide new perspectives of the treatments of early-stage MALT-type PPL.

## Conclusion

In conclusion, early-stage MALT-type PPL has an indolent course with a favorable long-term survival. In addition, neither surgery, chemotherapy nor a combination of surgery and chemotherapy can improve OS and CSS. In these circumstances, “watch and wait” may be a reasonable alternative. Large and well-designed randomized controlled trials are needed to confirm our results.

## Data availability statement

The datasets presented in this study can be found in online repositories. The names of the repository/repositories and accession number(s) can be found in the article/**Supplementary Material**.

## Ethics statement

Ethical approval was not provided for this study on human participants because the data of this study was extracted from the public SEER database and we have permission to the database. The patients/participants provided their written informed consent to participate in this study. Written informed consent was obtained from the individual(s) for the publication of any potentially identifiable images or data included in this article.

## Author contributions

HHL and KZ: concept, design, data acquisition, statistical analysis, and drafting of the manuscript. ZYP, JC and LCL: interpretation of data and review. JDM: concept, design, funding acquisition, supervision and editing. All authors contributed to the article and approved the submission.

## Funding

This work is supported by the 1.3.5 Project for Disciplines of Excellence (ZYJC18009), West China Hospital, Sichuan University to Dr. Mei.

## Acknowledgments

The authors appreciate the SEER program for granting access to the database.

## Conflict of interest

The authors declare that the research was conducted in the absence of any commercial or financial relationships that could be construed as a potential conflict of interest.

## Publisher’s note

All claims expressed in this article are solely those of the authors and do not necessarily represent those of their affiliated organizations, or those of the publisher, the editors and the reviewers. Any product that may be evaluated in this article, or claim that may be made by its manufacturer, is not guaranteed or endorsed by the publisher.
