# Supplementary material for: Surgery and chemotherapy cannot improve the survival of patients with early-stage mucosa-associated lymphoid tissue derived primary pulmonary lymphoma
Source: Front Oncol. 2022 Aug 23;12:965727. doi: 10.3389/fonc.2022.965727 (PMC9446888; doi:10.3389/fonc.2022.965727)
Supplement: Supplementary file 1 [file DataSheet_1.docx]

Supplementary Material

1. **Supplementary Tables**

Table S1 Characteristics between the None group and the Surgery alone group after PSM

| Variables |  | None | Surgery alone | P value |
| --- | --- | --- | --- | --- |
| Number |  | 259 | 259 |  |
| Age(years) |  | 68.61 ± 10.54 | 69.38 ± 12.26 | 0.440 |
| Sex | Female | 150 (57.9%) | 152 (58.7%) | 0.859 |
|  | Male | 109 (42.1%) | 107 (41.3%) |  |
| Race | White | 223 (86.1%) | 222 (85.7%) |  |
|  | black | 18 (6.9%) | 19 (7.3%) |  |
|  | Other | 18 (6.9%) | 18 (6.9%) |  |
| Marital status | Single | 41 (15.8%) | 29 (11.2%) | 0.223 |
|  | Married | 146 (56.4%) | 162 (62.5%) |  |
|  | Separated | 72 (27.8%) | 68 (26.3%) |  |
| Year of diagnosis | <2000 | 19 (7.3%) | 28 (10.8%) | 0.205 |
|  | 2000-2009 | 107 (41.3%) | 115 (44.4%) |  |
|  | ≥2010 | 133 (51.4%) | 116 (44.8%) |  |
| Primary site | Upper lobe | 93 (35.9%)) | 86 (33.2%) | 0.120 |
|  | Middle lobe | 30 (11.6%) | 41 (15.8%) |  |
|  | Lower lobe | 88 (34.0%)) | 101 (39.0%) |  |
|  | Main bronchus | 4 (1.5%) | 1 (0.4%) |  |
|  | NOS | 44 (17.0%) | 30 (11.6%) |  |
| Laterality | Unilateral | 251 (96.9%) | 249 (96.1%) | 0.631 |
|  | Bilateral | 8 (3.1%) | 10 (3.9%) |  |
| Stage | I | 223 (86.1%) | 225 (86.9%) | 0.797 |
|  | II | 36 (13.9%) | 34 (13.1%) |  |

NOS, not other specified.

Table S2 Characteristics between the None group and the Chemo alone group after PSM

| Variables |  | None | Chemo alone | P value |
| --- | --- | --- | --- | --- |
| Number |  | 142 | 142 |  |
| Age(years) |  | 68.09 ± 12.21 | 67.86 ± 12.02 | 0.876 |
| Sex | Female | 79 (55.6%) | 81 (57.0%) | 0.811 |
|  | Male | 63 (44.4%) | 61 (43.0%) |  |
| Race | White | 122 (85.9%) | 118 (83.1%) | 0.787 |
|  | black | 9 (6.3%) | 10 (7.0%) |  |
|  | Other | 11 (7.7%) | 14 (9.9%) |  |
| Marital status | Single | 25 (17.6%) | 21 (14.8%) | 0.554 |
|  | Married | 78 (54.9%) | 87 (61.3%) |  |
|  | Separated | 39 (27.5%) | 34 (23.9%) |  |
| Year of diagnosis | <2000 | 10 (7.0%) | 4 (2.8%) | 0.157 |
|  | 2000-2009 | 58 (40.8%) | 69 (48.6%) |  |
|  | ≥2010 | 74 (52.1%) | 69 (48.6%) |  |
| Primary site | Upper lobe | 34 (23.9%) | 43 (30.3%) | 0.103 |
|  | Middle lobe | 15 (10.6%) | 13 (9.2%) |  |
|  | Lower lobe | 56 (39.4%) | 36 (25.4%) |  |
|  | Main bronchus | 2 (1.4%) | 4 (2.8%) |  |
|  | NOS | 35 (24.6%) | 46 (32.4%) |  |
| Laterality | Unilateral | 124 (87.3%) | 124 (87.3%) | 1.000 |
|  | Bilateral | 18 (12.7%) | 18 (12.7%) |  |
| Stage | I | 112 (78.9%) | 112 (78.9%) | 1.000 |
|  | II | 30 (21.1%) | 30 (21.1%) |  |

NOS, not other specified.

Table S3 Characteristics between the None group and the Surgery + Chemo group after PSM

| Variables |  | None | Surgery + Chemo | P value |
| --- | --- | --- | --- | --- |
| Number |  | 66 | 66 |  |
| Age(years) |  | 64.36 ± 10.77 | 65.03 ± 12.74 | 0.746 |
| Sex | Female | 31 (47.0%) | 38 (57.6%) | 0.223 |
|  | Male | 35 (53.0%) | 28 (42.4%) |  |
| Race | White | 63 (95.5%) | 62 (93.9%) | 0.901 |
|  | black | 2 (3.0%) | 3 (3.0%) |  |
|  | Other | 1 (1.5%) | 1 (1.5%) |  |
| Marital status | Single | 11 (16.7%) | 9 (13.6%) | 0.655 |
|  | Married | 41 (62.1%) | 46 (69.7%) |  |
|  | Separated | 14 (21.2%) | 11 (16.7%) |  |
| Year of diagnosis | <2000 | 10 (15.2%) | 4 (6.1%) | 0.144 |
|  | 2000-2009 | 32 (48.5%) | 41 (62.1%) |  |
|  | ≥2010 | 24 (36.4%) | 21 (31.8%) |  |
| Primary site | Upper lobe | 13 (19.7%) | 14 (21.2%) | 0.998 |
|  | Middle lobe | 9 (13.6%) | 8 (12.1%) |  |
|  | Lower lobe | 20 (30.3%) | 19 (28.8%) |  |
|  | Main bronchus | 2 (3.0%) | 2 (3.0%) |  |
|  | NOS | 22 (33.3%) | 23 (34.8%) |  |
| Laterality | Unilateral | 53 (80.3%) | 53 (80.3%) | 1.00 |
|  | Bilateral | 13 (19.7%) | 13 (19.7%) |  |
| Stage | I | 53 (80.3%) | 55 (83.3%) | 0.652 |
|  | II | 13 (19.7%) | 11 (16.7%) |  |

NOS, not other specified.

Table S4 Characteristics between the Surgery alone group and the Chemo alone group after PSM

| Variables |  | Surgery alone | Chemo alone | P value |
| --- | --- | --- | --- | --- |
| Number |  | 138 | 138 |  |
| Age(years) |  | 67.78 ± 12.01 | 66.72 ± 12.97 | 0.483 |
| Sex | Female | 80 (58.0%) | 79 (57.2%) | 0.903 |
|  | Male | 58 (42.0%) | 59 (42.8%) |  |
| Race | White | 109 (79.0%) | 114 (82.6%) | 0.392 |
|  | black | 19 (13.8%) | 12 (8.7%) |  |
|  | Other | 10 (7.2%) | 12 (8.7%) |  |
| Marital status | Single | 22 (15.9%) | 23 (16.7%) | 0.993 |
|  | Married | 87 (63.0%) | 84 (60.9%) |  |
|  | Separated | 29 (21.0%) | 31 (22.5%) |  |
| Year of diagnosis | <2000 | 9 (6.5%) | 6 (4.3%) | 0.664 |
|  | 2000-2009 | 64 (46.4%) | 69 (50.0%) |  |
|  | ≥2010 | 65 (47.1%) | 63 (45.7%) |  |
| Primary site | Upper lobe | 38 (27.5%) | 47 (34.1%) | 0.067 |
|  | Middle lobe | 20 (14.5%) | 16 (11.6%) |  |
|  | Lower lobe | 55 (39.9%) | 36 (26.1%) |  |
|  | Main bronchus | 2 (1.4%) | 5 (3.6%) |  |
|  | NOS | 23 (16.7%) | 34 (24.6%) |  |
| Laterality | Unilateral | 131 (94.9%) | 127 (92.0%) | 0.329 |
|  | Bilateral | 7 (5.1%) | 11 (8.0%) |  |
| Stage | I | 98 (71.0%) | 101 (73.2%) | 0.687 |
|  | II | 40 (29.0%) | 37 (26.8%) |  |

NOS, not other specified.

Table S5 Characteristics between the Surgery alone group and the Surgery + Chemo group after PSM

| Variables |  | Surgery alone | Surgery + Chemo | P value |
| --- | --- | --- | --- | --- |
| Number |  | 63 | 63 |  |
| Age(years) |  | 63.92 ± 12.94 | 62.68 ± 12.05 | 0.579 |
| Sex | Female | 38 (60.3%) | 36 (57.1%) | 0.717 |
|  | Male | 25 (39.7%) | 27 (42.9%) |  |
| Race | White | 58 (92.1%) | 58 (92.1%) | 0.574 |
|  | black | 5 (7.9%) | 4 (6.3%) |  |
|  | Other | 0 (0.0%) | 1 (1.6%) |  |
| Marital status | Single | 13 (20.6%) | 9 (14.3%) | 0.584 |
|  | Married | 40 (63.5%) | 45 (71.4%) |  |
|  | Separated | 10 (15.9%) | 9 (14.3%) |  |
| Year of diagnosis | <2000 | 6 (9.5%) | 4 (6.3%) | 0.258 |
|  | 2000-2009 | 34 (54.0%) | 43 (68.3%) |  |
|  | ≥2010 | 23 (36.5%) | 16 (25.4%) |  |
| Primary site | Upper lobe | 21 (33.3%) | 15 (23.8%) | 0.524 |
|  | Middle lobe | 7 (11.1%) | 8 (12.7%) |  |
|  | Lower lobe | 18 (28.6%) | 19 (30.2%) |  |
|  | Main bronchus | 0 (0.0%) | 2 (3.2%) |  |
|  | NOS | 17 (27.0%) | 19 (30.2%) |  |
| Laterality | Unilateral | 57 (90.5%) | 55(87.3%) | 0.571 |
|  | Bilateral | 6 (9.5%) | 8 (12.7%) |  |
| Stage | I | 52 (82.5%) | 52 (82.5%) | 1.000 |
|  | II | 11 (17.5%) | 11 (17.5%) |  |

NOS, not other specified.

Table S6 | Characteristics between the Chemo alone group and the Surgery + Chemo group after PSM

| Variables |  | Chemo alone | Surgery + Chemo | P value |
| --- | --- | --- | --- | --- |
| Number |  | 72 | 72 |  |
| Age(years) |  | 62.29 ± 11.96 | 63.26 ± 11.30 | 0.617 |
| Sex | Female | 42 (58.3%) | 43 (59.7%) | 0.865 |
|  | Male | 30 (41.7%) | 29 (40.3%) |  |
| Race | White | 67 (93.1%) | 68 (94.4%) | 0.604 |
|  | black | 4 (5.6%) | 4 (5.6%) |  |
|  | Other | 1 (1.4%) | 0 (0.0%) |  |
| Marital status | Single | 9 (12.5%) | 11 (15.3%) | 0.442 |
|  | Married | 52 (72.2%) | 45 (62.5%) |  |
|  | Separated | 11 (15.3%) | 16 (22.2%) |  |
| Year of diagnosis | <2000 | 4 (5.6%) | 4 (5.6%) | 0.773 |
|  | 2000-2009 | 46 (63.9%) | 42 (58.3%) |  |
|  | ≥2010 | 22 (30.6%) | 26 (36.1%) |  |
| Primary site | Upper lobe | 15 (20.8%) | 20 (27.8%) | 0.870 |
|  | Middle lobe | 8 (11.1%) | 7 (9.7%) |  |
|  | Lower lobe | 19 (26.4%) | 15 (20.8%) |  |
|  | Main bronchus | 2 (2.8%) | 2 (2.8%) |  |
|  | NOS | 28 (38.9%) | 28 (38.9%) |  |
| Laterality | Unilateral | 55 (76.4%) | 61 (84.7%) | 0.206 |
|  | Bilateral | 17 (23.6%) | 11 (15.3%) |  |
| Stage | I | 59 (81.9%) | 57 (79.2%) | 0.674 |
|  | II | 13 (18.1%) | 15 (20.8%) |  |

NOS, not other specified.
